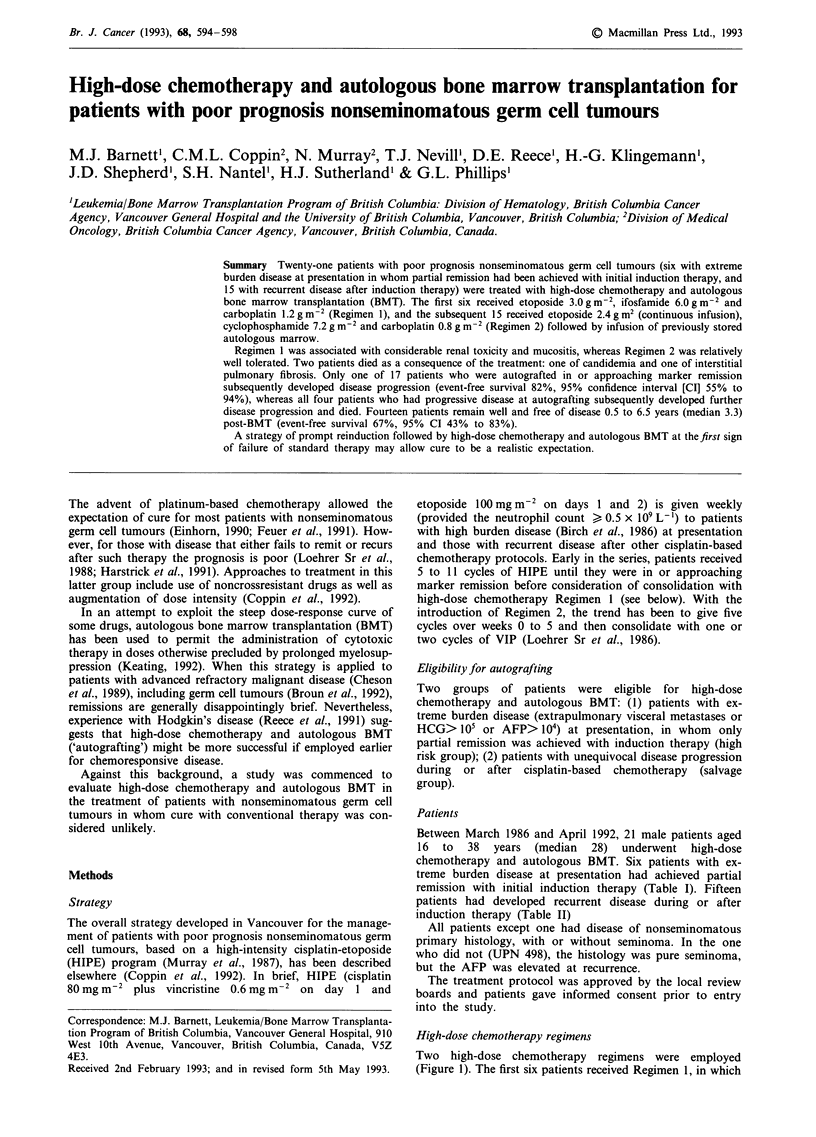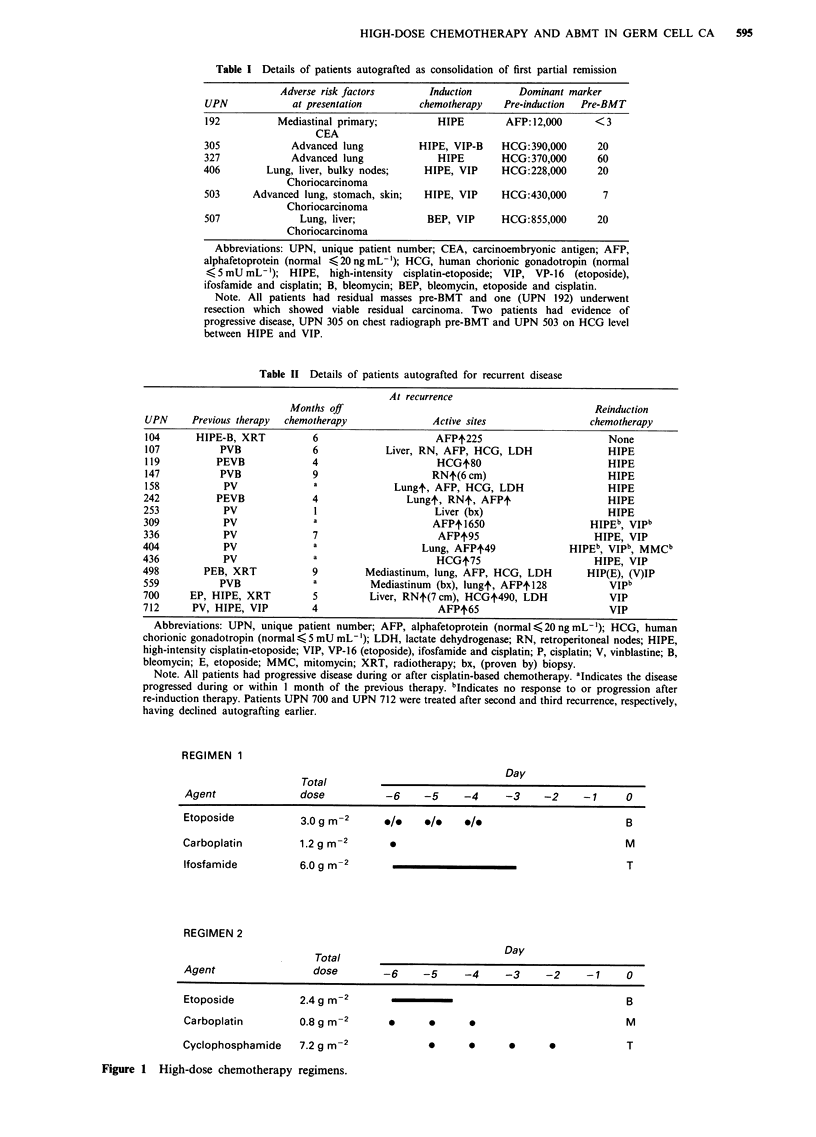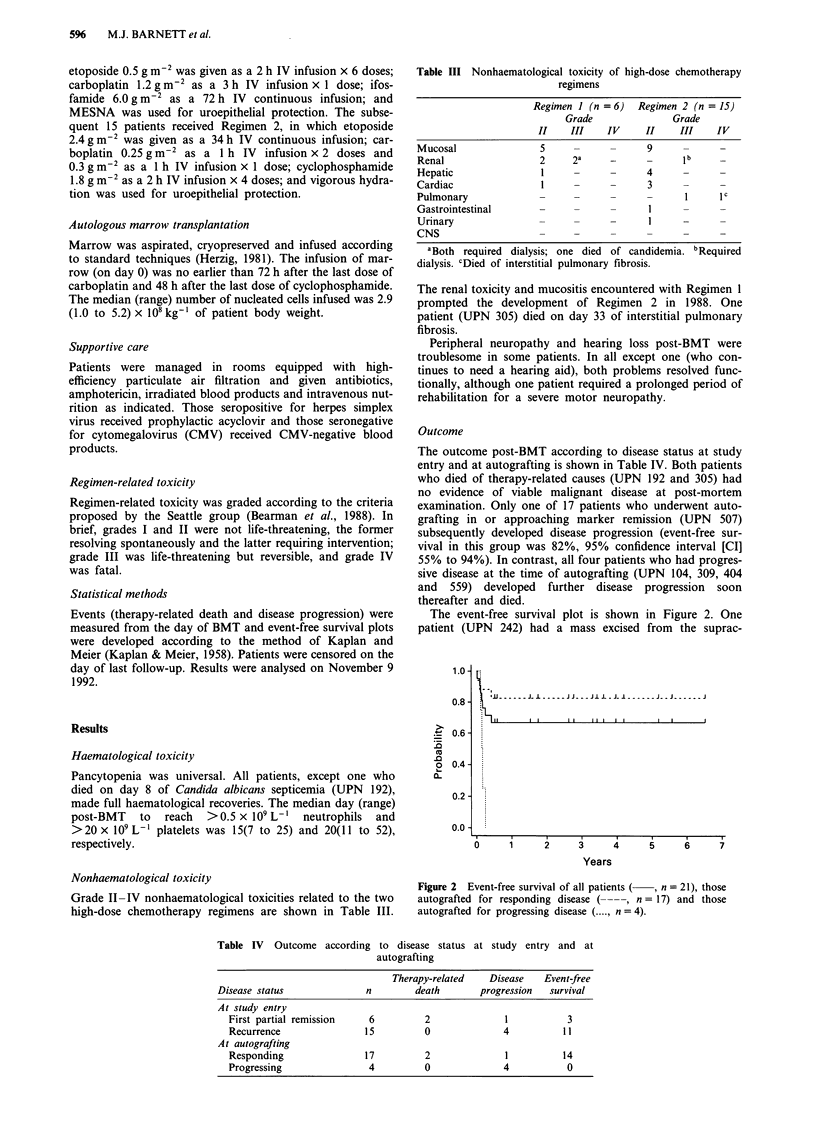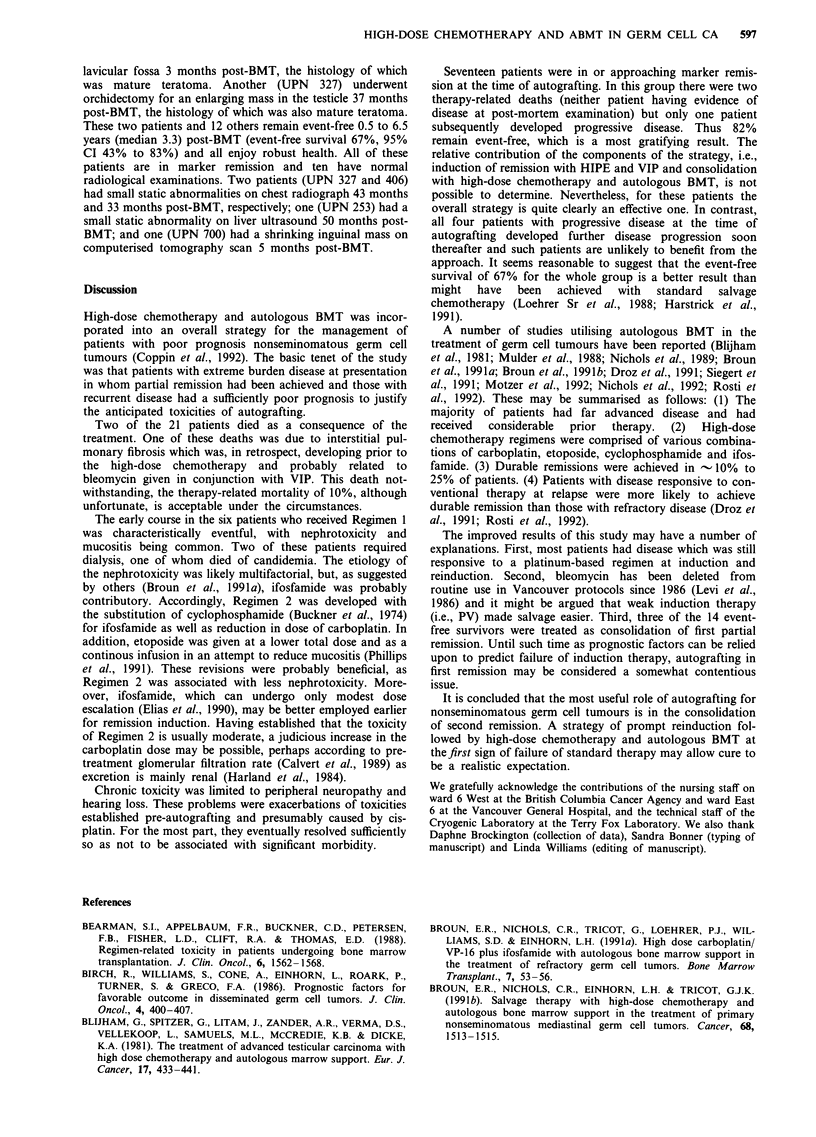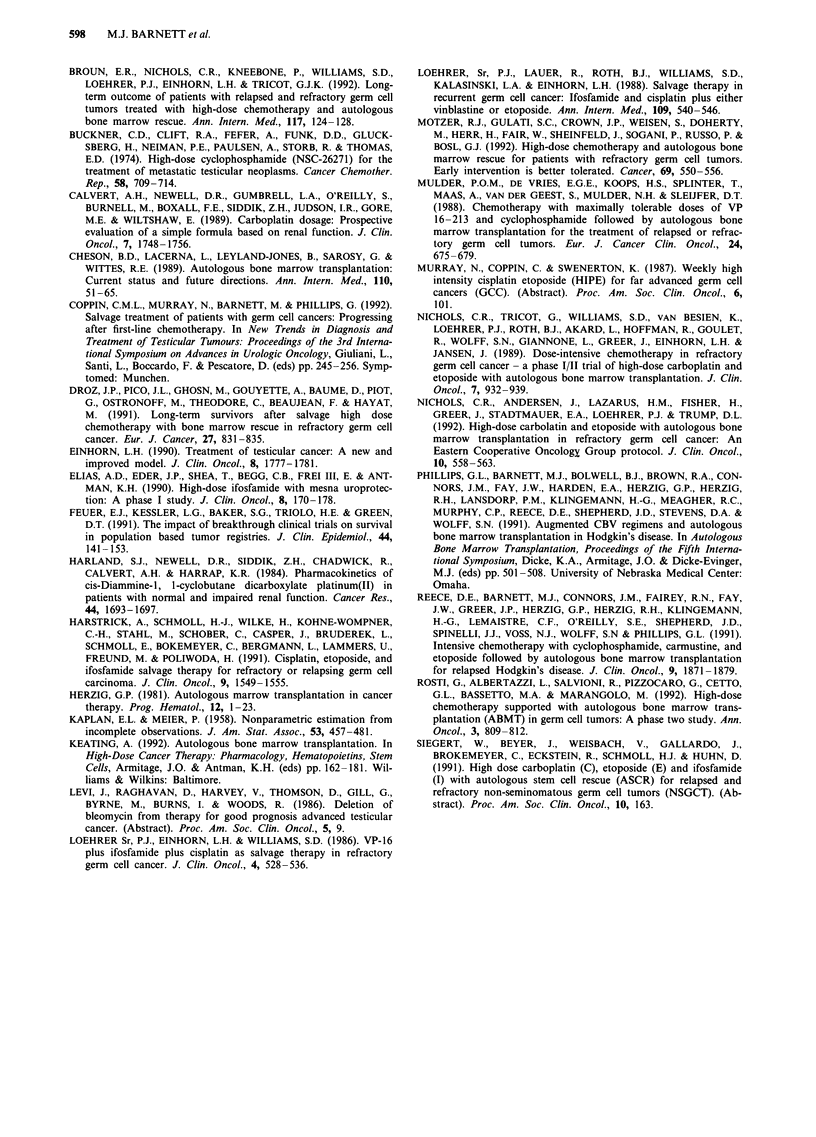# High-dose chemotherapy and autologous bone marrow transplantation for patients with poor prognosis nonseminomatous germ cell tumours.

**DOI:** 10.1038/bjc.1993.392

**Published:** 1993-09

**Authors:** M. J. Barnett, C. M. Coppin, N. Murray, T. J. Nevill, D. E. Reece, H. G. Klingemann, J. D. Shepherd, S. H. Nantel, H. J. Sutherland, G. L. Phillips

**Affiliations:** Leukemia/Bone Marrow Transplantation Program of British Columbia, Vancouver General Hospital, Canada.

## Abstract

Twenty-one patients with poor prognosis nonseminomatous germ cell tumours (six with extreme burden disease at presentation in whom partial remission had been achieved with initial induction therapy, and 15 with recurrent disease after induction therapy) were treated with high-dose chemotherapy and autologous bone marrow transplantation (BMT). The first six received etoposide 3.0 g m-2, ifosfamide 6.0 g m-2 and carboplatin 1.2 g m-2 (Regimen 1), and the subsequent 15 received etoposide 2.4 g m2 (continuous infusion), cyclophosphamide 7.2 g m-2 and carboplatin 0.8 g m-2 (Regimen 2) followed by infusion of previously stored autologous marrow. Regimen 1 was associated with considerable renal toxicity and mucositis, whereas Regimen 2 was relatively well tolerated. Two patients died as a consequence of the treatment: one of candidemia and one of interstitial pulmonary fibrosis. Only one of 17 patients who were autografted in or approaching marker remission subsequently developed disease progression (event-free survival 82%, 95% confidence interval [CI] 55% to 94%), whereas all four patients who had progressive disease at autografting subsequently developed further disease progression and died. Fourteen patients remain well and free of disease 0.5 to 6.5 years (median 3.3) post-BMT (event-free survival 67%, 95% CI 43% to 83%). A strategy of prompt reinduction followed by high-dose chemotherapy and autologous BMT at the first sign of failure of standard therapy may allow cure to be a realistic expectation.


Br. J. Cancer (1993), 68, 594-598 © Macmillan Press Ltd., 1993

### High-dose chemotherapy and autologous bone marrow transplantation for patients with poor prognosis nonseminomatous germ cell tumours

M.J. Barnett[1], C.M.L. Coppin[2], N. Murray[2], T.J. Nevill[1], D.E. Reece[1], H.-G. Klingemann[1], J.D. Shepherd[1], S.H. Nantel[1], H.J. Sutherland[1] & G.L. Phillips[1]

[1]*Leukemia/Bone Marrow Transplantation Program of British Columbia: Division of Hematology, British Columbia Cancer Agency, Vancouver General Hospital and the University of British Columbia, Vancouver, British Columbia;* [2]*Division of Medical Oncology, British Columbia Cancer Agency, Vancouver, British Columbia, Canada.*

**Summary** Twenty-one patients with poor prognosis nonseminomatous germ cell tumours (six with extreme burden disease at presentation in whom partial remission had been achieved with initial induction therapy, and 15 with recurrent disease after induction therapy) were treated with high-dose chemotherapy and autologous bone marrow transplantation (BMT). The first six received etoposide 3.0 g $m^{-2}$, ifosfamide 6.0 g $m^{-2}$ and carboplatin 1.2 g $m^{-2}$ (Regimen 1), and the subsequent 15 received etoposide 2.4 g $m^2$ (continuous infusion), cyclophosphamide 7.2 g $m^{-2}$ and carboplatin 0.8 g $m^{-2}$ (Regimen 2) followed by infusion of previously stored autologous marrow.

Regimen 1 was associated with considerable renal toxicity and mucositis, whereas Regimen 2 was relatively well tolerated. Two patients died as a consequence of the treatment: one of candidemia and one of interstitial pulmonary fibrosis. Only one of 17 patients who were autografted in or approaching marker remission subsequently developed disease progression (event-free survival 82%, 95% confidence interval [CI] 55% to 94%), whereas all four patients who had progressive disease at autografting subsequently developed further disease progression and died. Fourteen patients remain well and free of disease 0.5 to 6.5 years (median 3.3) post-BMT (event-free survival 67%, 95% CI 43% to 83%).

A strategy of prompt reinduction followed by high-dose chemotherapy and autologous BMT at the *first* sign of failure of standard therapy may allow cure to be a realistic expectation.

The advent of platinum-based chemotherapy allowed the expectation of cure for most patients with nonseminomatous germ cell tumours (Einhorn, 1990; Feuer *et al.*, 1991). However, for those with disease that either fails to remit or recurs after such therapy the prognosis is poor (Loehrer Sr *et al.*, 1988; Harstrick *et al.*, 1991). Approaches to treatment in this latter group include use of noncrossresistant drugs as well as augmentation of dose intensity (Coppin *et al.*, 1992).

In an attempt to exploit the steep dose-response curve of some drugs, autologous bone marrow transplantation (BMT) has been used to permit the administration of cytotoxic therapy in doses otherwise precluded by prolonged myelosuppression (Keating, 1992). When this strategy is applied to patients with advanced refractory malignant disease (Cheson *et al.*, 1989), including germ cell tumours (Broun *et al.*, 1992), remissions are generally disappointingly brief. Nevertheless, experience with Hodgkin's disease (Reece *et al.*, 1991) suggests that high-dose chemotherapy and autologous BMT ('autografting') might be more successful if employed earlier for chemoresponsive disease.

Against this background, a study was commenced to evaluate high-dose chemotherapy and autologous BMT in the treatment of patients with nonseminomatous germ cell tumours in whom cure with conventional therapy was considered unlikely.

### Methods

### *Strategy*

The overall strategy developed in Vancouver for the management of patients with poor prognosis nonseminomatous germ cell tumours, based on a high-intensity cisplatin-etoposide (HIPE) program (Murray *et al.*, 1987), has been described elsewhere (Coppin *et al.*, 1992). In brief, HIPE (cisplatin 80 mg $m^{-2}$ plus vincristine 0.6 mg $m^{-2}$ on day 1 and etoposide 100 mg $m^{-2}$ on days 1 and 2) is given weekly (provided the neutrophil count $\geqslant 0.5 \times 10^9 L^{-1}$) to patients with high burden disease (Birch *et al.*, 1986) at presentation and those with recurrent disease after other cisplatin-based chemotherapy protocols. Early in the series, patients received 5 to 11 cycles of HIPE until they were in or approaching marker remission before consideration of consolidation with high-dose chemotherapy Regimen 1 (see below). With the introduction of Regimen 2, the trend has been to give five cycles over weeks 0 to 5 and then consolidate with one or two cycles of VIP (Loehrer Sr *et al.*, 1986).

### *Eligibility for autografting*

Two groups of patients were eligible for high-dose chemotherapy and autologous BMT: (1) patients with extreme burden disease (extrapulmonary visceral metastases or HCG$>10^5$ or AFP$>10^4$) at presentation, in whom only partial remission was achieved with induction therapy (high risk group); (2) patients with unequivocal disease progression during or after cisplatin-based chemotherapy (salvage group).

### *Patients*

Between March 1986 and April 1992, 21 male patients aged 16 to 38 years (median 28) underwent high-dose chemotherapy and autologous BMT. Six patients with extreme burden disease at presentation had achieved partial remission with initial induction therapy (Table I). Fifteen patients had developed recurrent disease during or after induction therapy (Table II)

All patients except one had disease of nonseminomatous primary histology, with or without seminoma. In the one who did not (UPN 498), the histology was pure seminoma, but the AFP was elevated at recurrence.

The treatment protocol was approved by the local review boards and patients gave informed consent prior to entry into the study.

### *High-dose chemotherapy regimens*

Two high-dose chemotherapy regimens were employed (Figure 1). The first six patients received Regimen 1, in which

Correspondence: M.J. Barnett, Leukemia/Bone Marrow Transplantation Program of British Columbia, Vancouver General Hospital, 910 West 10th Avenue, Vancouver, British Columbia, Canada, V5Z 4E3.

Received 2nd February 1993; and in revised form 5th May 1993.

**Table I** Details of patients autografted as consolidation of first partial remission

| UPN | Adverse risk factors at presentation | Induction chemotherapy | Dominant marker Pre-induction | Pre-BMT |
|---|---|---|---|---|
| 192 | Mediastinal primary; CEA | HIPE | AFP:12,000 | <3 |
| 305 | Advanced lung | HIPE, VIP-B | HCG:390,000 | 20 |
| 327 | Advanced lung | HIPE | HCG:370,000 | 60 |
| 406 | Lung, liver, bulky nodes; Choriocarcinoma | HIPE, VIP | HCG:228,000 | 20 |
| 503 | Advanced lung, stomach, skin; Choriocarcinoma | HIPE, VIP | HCG:430,000 | 7 |
| 507 | Lung, liver; Choriocarcinoma | BEP, VIP | HCG:855,000 | 20 |

Abbreviations: UPN, unique patient number; CEA, carcinoembryonic antigen; AFP, alphafetoprotein (normal $\leqslant 20\,ng\,mL^{-1}$); HCG, human chorionic gonadotropin (normal $\leqslant 5\,mU\,mL^{-1}$); HIPE, high-intensity cisplatin-etoposide; VIP, VP-16 (etoposide), ifosfamide and cisplatin; B, bleomycin; BEP, bleomycin, etoposide and cisplatin.

Note. All patients had residual masses pre-BMT and one (UPN 192) underwent resection which showed viable residual carcinoma. Two patients had evidence of progressive disease, UPN 305 on chest radiograph pre-BMT and UPN 503 on HCG level between HIPE and VIP.

**Table II** Details of patients autografted for recurrent disease

| UPN | Previous therapy | At recurrence: Months off chemotherapy | Active sites | Reinduction chemotherapy |
|---|---|---|---|---|
| 104 | HIPE-B, XRT | 6 | AFP↑225 | None |
| 107 | PVB | 6 | Liver, RN, AFP, HCG, LDH | HIPE |
| 119 | PEVB | 4 | HCG↑80 | HIPE |
| 147 | PVB | 9 | RN↑(6 cm) | HIPE |
| 158 | PV | a | Lung↑, AFP, HCG, LDH | HIPE |
| 242 | PEVB | 4 | Lung↑, RN↑, AFP↑ | HIPE |
| 253 | PV | 1 | Liver (bx) | HIPE |
| 309 | PV | a | AFP↑1650 | HIPE[b], VIP[b] |
| 336 | PV | 7 | AFP↑95 | HIPE, VIP |
| 404 | PV | a | Lung, AFP↑49 | HIPE[b], VIP[b], MMC[b] |
| 436 | PV | a | HCG↑75 | HIPE, VIP |
| 498 | PEB, XRT | 9 | Mediastinum, lung, AFP, HCG, LDH | HIP(E), (V)IP |
| 559 | PVB | a | Mediastinum (bx), lung↑, AFP↑128 | VIP[b] |
| 700 | EP, HIPE, XRT | 5 | Liver, RN↑(7 cm), HCG↑490, LDH | VIP |
| 712 | PV, HIPE, VIP | 4 | AFP↑65 | VIP |

Abbreviations: UPN, unique patient number; AFP, alphafetoprotein (normal $\leqslant 20\,ng\,mL^{-1}$); HCG, human chorionic gonadotropin (normal $\leqslant 5\,mU\,mL^{-1}$); LDH, lactate dehydrogenase; RN, retroperitoneal nodes; HIPE, high-intensity cisplatin-etoposide; VIP, VP-16 (etoposide), ifosfamide and cisplatin; P, cisplatin; V, vinblastine; B, bleomycin; E, etoposide; MMC, mitomycin; XRT, radiotherapy; bx, (proven by) biopsy.

Note. All patients had progressive disease during or after cisplatin-based chemotherapy. [a]Indicates the disease progressed during or within 1 month of the previous therapy. [b]Indicates no response to or progression after re-induction therapy. Patients UPN 700 and UPN 712 were treated after second and third recurrence, respectively, having declined autografting earlier.

**Figure 1** High-dose chemotherapy regimens.

etoposide 0.5 g $m^{-2}$ was given as a 2 h IV infusion × 6 doses; carboplatin 1.2 g $m^{-2}$ as a 3 h IV infusion × 1 dose; ifosfamide 6.0 g $m^{-2}$ as a 72 h IV continuous infusion; and MESNA was used for uroepithelial protection. The subsequent 15 patients received Regimen 2, in which etoposide 2.4 g $m^{-2}$ was given as a 34 h IV continuous infusion; carboplatin 0.25 g $m^{-2}$ as a 1 h IV infusion × 2 doses and 0.3 g $m^{-2}$ as a 1 h IV infusion × 1 dose; cyclophosphamide 1.8 g $m^{-2}$ as a 2 h IV infusion × 4 doses; and vigorous hydration was used for uroepithelial protection.

### *Autologous marrow transplantation*

Marrow was aspirated, cryopreserved and infused according to standard techniques (Herzig, 1981). The infusion of marrow (on day 0) was no earlier than 72 h after the last dose of carboplatin and 48 h after the last dose of cyclophosphamide. The median (range) number of nucleated cells infused was 2.9 (1.0 to 5.2) × $10^8$ $kg^{-1}$ of patient body weight.

### *Supportive care*

Patients were managed in rooms equipped with high-efficiency particulate air filtration and given antibiotics, amphotericin, irradiated blood products and intravenous nutrition as indicated. Those seropositive for herpes simplex virus received prophylactic acyclovir and those seronegative for cytomegalovirus (CMV) received CMV-negative blood products.

### *Regimen-related toxicity*

Regimen-related toxicity was graded according to the criteria proposed by the Seattle group (Bearman *et al.*, 1988). In brief, grades I and II were not life-threatening, the former resolving spontaneously and the latter requiring intervention; grade III was life-threatening but reversible, and grade IV was fatal.

### *Statistical methods*

Events (therapy-related death and disease progression) were measured from the day of BMT and event-free survival plots were developed according to the method of Kaplan and Meier (Kaplan & Meier, 1958). Patients were censored on the day of last follow-up. Results were analysed on November 9 1992.

### Results

### *Haematological toxicity*

Pancytopenia was universal. All patients, except one who died on day 8 of *Candida albicans* septicemia (UPN 192), made full haematological recoveries. The median day (range) post-BMT to reach $>0.5 \times 10^9$ $L^{-1}$ neutrophils and $>20 \times 10^9$ $L^{-1}$ platelets was 15(7 to 25) and 20(11 to 52), respectively.

### *Nonhaematological toxicity*

Grade II–IV nonhaematological toxicities related to the two high-dose chemotherapy regimens are shown in Table III.

**Table III** Nonhaematological toxicity of high-dose chemotherapy regimens

| | *Regimen 1 (n = 6)* Grade | | | *Regimen 2 (n = 15)* Grade | | |
|---|---|---|---|---|---|---|
| | *II* | *III* | *IV* | *II* | *III* | *IV* |
| Mucosal | 5 | – | – | 9 | – | – |
| Renal | 2 | 2[a] | – | – | 1[b] | – |
| Hepatic | 1 | – | – | 4 | – | – |
| Cardiac | 1 | – | – | 3 | – | – |
| Pulmonary | – | – | – | – | 1 | 1[c] |
| Gastrointestinal | – | – | – | 1 | – | – |
| Urinary | – | – | – | 1 | – | – |
| CNS | – | – | – | – | – | – |

[a]Both required dialysis; one died of candidemia. [b]Required dialysis. [c]Died of interstitial pulmonary fibrosis.

The renal toxicity and mucositis encountered with Regimen 1 prompted the development of Regimen 2 in 1988. One patient (UPN 305) died on day 33 of interstitial pulmonary fibrosis.

Peripheral neuropathy and hearing loss post-BMT were troublesome in some patients. In all except one (who continues to need a hearing aid), both problems resolved functionally, although one patient required a prolonged period of rehabilitation for a severe motor neuropathy.

### *Outcome*

The outcome post-BMT according to disease status at study entry and at autografting is shown in Table IV. Both patients who died of therapy-related causes (UPN 192 and 305) had no evidence of viable malignant disease at post-mortem examination. Only one of 17 patients who underwent autografting in or approaching marker remission (UPN 507) subsequently developed disease progression (event-free survival in this group was 82%, 95% confidence interval [CI] 55% to 94%). In contrast, all four patients who had progressive disease at the time of autografting (UPN 104, 309, 404 and 559) developed further disease progression soon thereafter and died.

The event-free survival plot is shown in Figure 2. One patient (UPN 242) had a mass excised from the suprac-

**Figure 2** Event-free survival of all patients (——, $n = 21$), those autografted for responding disease (– – – –, $n = 17$) and those autografted for progressing disease (....., $n = 4$).

**Table IV** Outcome according to disease status at study entry and at autografting

| *Disease status* | *n* | *Therapy-related death* | *Disease progression* | *Event-free survival* |
|---|---|---|---|---|
| *At study entry* | | | | |
| First partial remission | 6 | 2 | 1 | 3 |
| Recurrence | 15 | 0 | 4 | 11 |
| *At autografting* | | | | |
| Responding | 17 | 2 | 1 | 14 |
| Progressing | 4 | 0 | 4 | 0 |

lavicular fossa 3 months post-BMT, the histology of which was mature teratoma. Another (UPN 327) underwent orchidectomy for an enlarging mass in the testicle 37 months post-BMT, the histology of which was also mature teratoma. These two patients and 12 others remain event-free 0.5 to 6.5 years (median 3.3) post-BMT (event-free survival 67%, 95% CI 43% to 83%) and all enjoy robust health. All of these patients are in marker remission and ten have normal radiological examinations. Two patients (UPN 327 and 406) had small static abnormalities on chest radiograph 43 months and 33 months post-BMT, respectively; one (UPN 253) had a small static abnormality on liver ultrasound 50 months post-BMT; and one (UPN 700) had a shrinking inguinal mass on computerised tomography scan 5 months post-BMT.

### Discussion

High-dose chemotherapy and autologous BMT was incorporated into an overall strategy for the management of patients with poor prognosis nonseminomatous germ cell tumours (Coppin *et al.*, 1992). The basic tenet of the study was that patients with extreme burden disease at presentation in whom partial remission had been achieved and those with recurrent disease had a sufficiently poor prognosis to justify the anticipated toxicities of autografting.

Two of the 21 patients died as a consequence of the treatment. One of these deaths was due to interstitial pulmonary fibrosis which was, in retrospect, developing prior to the high-dose chemotherapy and probably related to bleomycin given in conjunction with VIP. This death notwithstanding, the therapy-related mortality of 10%, although unfortunate, is acceptable under the circumstances.

The early course in the six patients who received Regimen 1 was characteristically eventful, with nephrotoxicity and mucositis being common. Two of these patients required dialysis, one of whom died of candidemia. The etiology of the nephrotoxicity was likely multifactorial, but, as suggested by others (Broun *et al.*, 1991*a*), ifosfamide was probably contributory. Accordingly, Regimen 2 was developed with the substitution of cyclophosphamide (Buckner *et al.*, 1974) for ifosfamide as well as reduction in dose of carboplatin. In addition, etoposide was given at a lower total dose and as a continous infusion in an attempt to reduce mucositis (Phillips *et al.*, 1991). These revisions were probably beneficial, as Regimen 2 was associated with less nephrotoxicity. Moreover, ifosfamide, which can undergo only modest dose escalation (Elias *et al.*, 1990), may be better employed earlier for remission induction. Having established that the toxicity of Regimen 2 is usually moderate, a judicious increase in the carboplatin dose may be possible, perhaps according to pretreatment glomerular filtration rate (Calvert *et al.*, 1989) as excretion is mainly renal (Harland *et al.*, 1984).

Chronic toxicity was limited to peripheral neuropathy and hearing loss. These problems were exacerbations of toxicities established pre-autografting and presumably caused by cisplatin. For the most part, they eventually resolved sufficiently so as not to be associated with significant morbidity.

Seventeen patients were in or approaching marker remission at the time of autografting. In this group there were two therapy-related deaths (neither patient having evidence of disease at post-mortem examination) but only one patient subsequently developed progressive disease. Thus 82% remain event-free, which is a most gratifying result. The relative contribution of the components of the strategy, i.e., induction of remission with HIPE and VIP and consolidation with high-dose chemotherapy and autologous BMT, is not possible to determine. Nevertheless, for these patients the overall strategy is quite clearly an effective one. In contrast, all four patients with progressive disease at the time of autografting developed further disease progression soon thereafter and such patients are unlikely to benefit from the approach. It seems reasonable to suggest that the event-free survival of 67% for the whole group is a better result than might have been achieved with standard salvage chemotherapy (Loehrer Sr *et al.*, 1988; Harstrick *et al.*, 1991).

A number of studies utilising autologous BMT in the treatment of germ cell tumours have been reported (Blijham *et al.*, 1981; Mulder *et al.*, 1988; Nichols *et al.*, 1989; Broun *et al.*, 1991*a*; Broun *et al.*, 1991*b*; Droz *et al.*, 1991; Siegert *et al.*, 1991; Motzer *et al.*, 1992; Nichols *et al.*, 1992; Rosti *et al.*, 1992). These may be summarised as follows: (1) The majority of patients had far advanced disease and had received considerable prior therapy. (2) High-dose chemotherapy regimens were comprised of various combinations of carboplatin, etoposide, cyclophosphamide and ifosfamide. (3) Durable remissions were achieved in $\sim$10% to 25% of patients. (4) Patients with disease responsive to conventional therapy at relapse were more likely to achieve durable remission than those with refractory disease (Droz *et al.*, 1991; Rosti *et al.*, 1992).

The improved results of this study may have a number of explanations. First, most patients had disease which was still responsive to a platinum-based regimen at induction and reinduction. Second, bleomycin has been deleted from routine use in Vancouver protocols since 1986 (Levi *et al.*, 1986) and it might be argued that weak induction therapy (i.e., PV) made salvage easier. Third, three of the 14 event-free survivors were treated as consolidation of first partial remission. Until such time as prognostic factors can be relied upon to predict failure of induction therapy, autografting in first remission may be considered a somewhat contentious issue.

It is concluded that the most useful role of autografting for nonseminomatous germ cell tumours is in the consolidation of second remission. A strategy of prompt reinduction followed by high-dose chemotherapy and autologous BMT at the *first* sign of failure of standard therapy may allow cure to be a realistic expectation.

We gratefully acknowledge the contributions of the nursing staff on ward 6 West at the British Columbia Cancer Agency and ward East 6 at the Vancouver General Hospital, and the technical staff of the Cryogenic Laboratory at the Terry Fox Laboratory. We also thank Daphne Brockington (collection of data), Sandra Bonner (typing of manuscript) and Linda Williams (editing of manuscript).

### References

BEARMAN, S.I., APPELBAUM, F.R., BUCKNER, C.D., PETERSEN, F.B., FISHER, L.D., CLIFT, R.A. & THOMAS, E.D. (1988). Regimen-related toxicity in patients undergoing bone marrow transplantation. *J. Clin. Oncol.*, **6**, 1562–1568.

BIRCH, R., WILLIAMS, S., CONE, A., EINHORN, L., ROARK, P., TURNER, S. & GRECO, F.A. (1986). Prognostic factors for favorable outcome in disseminated germ cell tumors. *J. Clin. Oncol.*, **4**, 400–407.

BLIJHAM, G., SPITZER, G., LITAM, J., ZANDER, A.R., VERMA, D.S., VELLEKOOP, L., SAMUELS, M.L., MCCREDIE, K.B. & DICKE, K.A. (1981). The treatment of advanced testicular carcinoma with high dose chemotherapy and autologous marrow support. *Eur. J. Cancer*, **17**, 433–441.

BROUN, E.R., NICHOLS, C.R., TRICOT, G., LOEHRER, P.J., WILLIAMS, S.D. & EINHORN, L.H. (1991*a*). High dose carboplatin/VP-16 plus ifosfamide with autologous bone marrow support in the treatment of refractory germ cell tumors. *Bone Marrow Transplant.*, **7**, 53–56.

BROUN, E.R., NICHOLS, C.R., EINHORN, L.H. & TRICOT, G.J.K. (1991*b*). Salvage therapy with high-dose chemotherapy and autologous bone marrow support in the treatment of primary nonseminomatous mediastinal germ cell tumors. *Cancer*, **68**, 1513–1515.

BROUN, E.R., NICHOLS, C.R., KNEEBONE, P., WILLIAMS, S.D., LOEHRER, P.J., EINHORN, L.H. & TRICOT, G.J.K. (1992). Long-term outcome of patients with relapsed and refractory germ cell tumors treated with high-dose chemotherapy and autologous bone marrow rescue. *Ann. Intern. Med.*, **117**, 124–128.

BUCKNER, C.D., CLIFT, R.A., FEFER, A., FUNK, D.D., GLUCKSBERG, H., NEIMAN, P.E., PAULSEN, A., STORB, R. & THOMAS, E.D. (1974). High-dose cyclophosphamide (NSC-26271) for the treatment of metastatic testicular neoplasms. *Cancer Chemother. Rep.*, **58**, 709–714.

CALVERT, A.H., NEWELL, D.R., GUMBRELL, L.A., O'REILLY, S., BURNELL, M., BOXALL, F.E., SIDDIK, Z.H., JUDSON, I.R., GORE, M.E. & WILTSHAW, E. (1989). Carboplatin dosage: Prospective evaluation of a simple formula based on renal function. *J. Clin. Oncol.*, **7**, 1748–1756.

CHESON, B.D., LACERNA, L., LEYLAND-JONES, B., SAROSY, G. & WITTES, R.E. (1989). Autologous bone marrow transplantation: Current status and future directions. *Ann. Intern. Med.*, **110**, 51–65.

COPPIN, C.M.L., MURRAY, N., BARNETT, M. & PHILLIPS, G. (1992). Salvage treatment of patients with germ cell cancers: Progressing after first-line chemotherapy. In *New Trends in Diagnosis and Treatment of Testicular Tumours: Proceedings of the 3rd International Symposium on Advances in Urologic Oncology*, Giuliani, L., Santi, L., Boccardo, F. & Pescatore, D. (eds) pp. 245–256. Symptomed: Munchen.

DROZ, J.P., PICO, J.L., GHOSN, M., GOUYETTE, A., BAUME, D., PIOT, G., OSTRONOFF, M., THEODORE, C., BEAUJEAN, F. & HAYAT, M. (1991). Long-term survivors after salvage high dose chemotherapy with bone marrow rescue in refractory germ cell cancer. *Eur. J. Cancer*, **27**, 831–835.

EINHORN, L.H. (1990). Treatment of testicular cancer: A new and improved model. *J. Clin. Oncol.*, **8**, 1777–1781.

ELIAS, A.D., EDER, J.P., SHEA, T., BEGG, C.B., FREI III, E. & ANTMAN, K.H. (1990). High-dose ifosfamide with mesna uroprotection: A phase I study. *J. Clin. Oncol.*, **8**, 170–178.

FEUER, E.J., KESSLER, L.G., BAKER, S.G., TRIOLO, H.E. & GREEN, D.T. (1991). The impact of breakthrough clinical trials on survival in population based tumor registries. *J. Clin. Epidemiol.*, **44**, 141–153.

HARLAND, S.J., NEWELL, D.R., SIDDIK, Z.H., CHADWICK, R., CALVERT, A.H. & HARRAP, K.R. (1984). Pharmacokinetics of cis-Diammine-1, 1-cyclobutane dicarboxylate platinum(II) in patients with normal and impaired renal function. *Cancer Res.*, **44**, 1693–1697.

HARSTRICK, A., SCHMOLL, H.-J., WILKE, H., KOHNE-WOMPNER, C.-H., STAHL, M., SCHOBER, C., CASPER, J., BRUDEREK, L., SCHMOLL, E., BOKEMEYER, C., BERGMANN, L., LAMMERS, U., FREUND, M. & POLIWODA, H. (1991). Cisplatin, etoposide, and ifosfamide salvage therapy for refractory or relapsing germ cell carcinoma. *J. Clin. Oncol.*, **9**, 1549–1555.

HERZIG, G.P. (1981). Autologous marrow transplantation in cancer therapy. *Prog. Hematol.*, **12**, 1–23.

KAPLAN, E.L. & MEIER, P. (1958). Nonparametric estimation from incomplete observations. *J. Am. Stat. Assoc.*, **53**, 457–481.

KEATING, A. (1992). Autologous bone marrow transplantation. In *High-Dose Cancer Therapy: Pharmacology, Hematopoietins, Stem Cells*, Armitage, J.O. & Antman, K.H. (eds) pp. 162–181. Williams & Wilkins: Baltimore.

LEVI, J., RAGHAVAN, D., HARVEY, V., THOMSON, D., GILL, G., BYRNE, M., BURNS, I. & WOODS, R. (1986). Deletion of bleomycin from therapy for good prognosis advanced testicular cancer. (Abstract). *Proc. Am. Soc. Clin. Oncol.*, **5**, 9.

LOEHRER Sr, P.J., EINHORN, L.H. & WILLIAMS, S.D. (1986). VP-16 plus ifosfamide plus cisplatin as salvage therapy in refractory germ cell cancer. *J. Clin. Oncol.*, **4**, 528–536.

LOEHRER, Sr, P.J., LAUER, R., ROTH, B.J., WILLIAMS, S.D., KALASINSKI, L.A. & EINHORN, L.H. (1988). Salvage therapy in recurrent germ cell cancer: Ifosfamide and cisplatin plus either vinblastine or etoposide. *Ann. Intern. Med.*, **109**, 540–546.

MOTZER, R.J., GULATI, S.C., CROWN, J.P., WEISEN, S., DOHERTY, M., HERR, H., FAIR, W., SHEINFELD, J., SOGANI, P., RUSSO, P. & BOSL, G.J. (1992). High-dose chemotherapy and autologous bone marrow rescue for patients with refractory germ cell tumors. Early intervention is better tolerated. *Cancer*, **69**, 550–556.

MULDER, P.O.M., DE VRIES, E.G.E., KOOPS, H.S., SPLINTER, T., MAAS, A., VAN DER GEEST, S., MULDER, N.H. & SLEIJFER, D.T. (1988). Chemotherapy with maximally tolerable doses of VP 16–213 and cyclophosphamide followed by autologous bone marrow transplantation for the treatment of relapsed or refractory germ cell tumors. *Eur. J. Cancer Clin. Oncol.*, **24**, 675–679.

MURRAY, N., COPPIN, C. & SWENERTON, K. (1987). Weekly high intensity cisplatin etoposide (HIPE) for far advanced germ cell cancers (GCC). (Abstract). *Proc. Am. Soc. Clin. Oncol.*, **6**, 101.

NICHOLS, C.R., TRICOT, G., WILLIAMS, S.D., VAN BESIEN, K., LOEHRER, P.J., ROTH, B.J., AKARD, L., HOFFMAN, R., GOULET, R., WOLFF, S.N., GIANNONE, L., GREER, J., EINHORN, L.H. & JANSEN, J. (1989). Dose-intensive chemotherapy in refractory germ cell cancer – a phase I/II trial of high-dose carboplatin and etoposide with autologous bone marrow transplantation. *J. Clin. Oncol.*, **7**, 932–939.

NICHOLS, C.R., ANDERSEN, J., LAZARUS, H.M., FISHER, H., GREER, J., STADTMAUER, E.A., LOEHRER, P.J. & TRUMP, D.L. (1992). High-dose carbolatin and etoposide with autologous bone marrow transplantation in refractory germ cell cancer: An Eastern Cooperative Oncology Group protocol. *J. Clin. Oncol.*, **10**, 558–563.

PHILLIPS, G.L., BARNETT, M.J., BOLWELL, B.J., BROWN, R.A., CONNORS, J.M., FAY, J.W., HARDEN, E.A., HERZIG, G.P., HERZIG, R.H., LANSDORP, P.M., KLINGEMANN, H.-G., MEAGHER, R.C., MURPHY, C.P., REECE, D.E., SHEPHERD, J.D., STEVENS, D.A. & WOLFF, S.N. (1991). Augmented CBV regimens and autologous bone marrow transplantation in Hodgkin's disease. In *Autologous Bone Marrow Transplantation, Proceedings of the Fifth International Symposium*, Dicke, K.A., Armitage, J.O. & Dicke-Evinger, M.J. (eds) pp. 501–508. University of Nebraska Medical Center: Omaha.

REECE, D.E., BARNETT, M.J., CONNORS, J.M., FAIREY, R.N., FAY, J.W., GREER, J.P., HERZIG, G.P., HERZIG, R.H., KLINGEMANN, H.-G., LEMAISTRE, C.F., O'REILLY, S.E., SHEPHERD, J.D., SPINELLI, J.J., VOSS, N.J., WOLFF, S.N & PHILLIPS, G.L. (1991). Intensive chemotherapy with cyclophosphamide, carmustine, and etoposide followed by autologous bone marrow transplantation for relapsed Hodgkin's disease. *J. Clin. Oncol.*, **9**, 1871–1879.

ROSTI, G., ALBERTAZZI, L., SALVIONI, R., PIZZOCARO, G., CETTO, G.L., BASSETTO, M.A. & MARANGOLO, M. (1992). High-dose chemotherapy supported with autologous bone marrow transplantation (ABMT) in germ cell tumors: A phase two study. *Ann. Oncol.*, **3**, 809–812.

SIEGERT, W., BEYER, J., WEISBACH, V., GALLARDO, J., BROKEMEYER, C., ECKSTEIN, R., SCHMOLL, H.J. & HUHN, D. (1991). High dose carboplatin (C), etoposide (E) and ifosfamide (I) with autologous stem cell rescue (ASCR) for relapsed and refractory non-seminomatous germ cell tumors (NSGCT). (Abstract). *Proc. Am. Soc. Clin. Oncol.*, **10**, 163.